# Nanocomposite Organogel for Art Conservation—A Novel Wax Resin Removal System

**DOI:** 10.1021/acsami.3c00321

**Published:** 2023-05-03

**Authors:** Klaudia Kaniewska, Elżbieta Pilecka-Pietrusińska, Marcin Karbarz

**Affiliations:** † Faculty of Chemistry, Biological and Chemical Research Center, University of Warsaw, 101 Żwirki i Wigury Av., PL, 02-089 Warsaw, Poland; ‡ National Museum in Warsaw, 3 Aleje Jerozolimskie Av., 00-495 Warsaw, Poland

**Keywords:** organogel, nanocomposite gel, Dutch Method, art conservation, gel for restoration, conservation of paintings

## Abstract

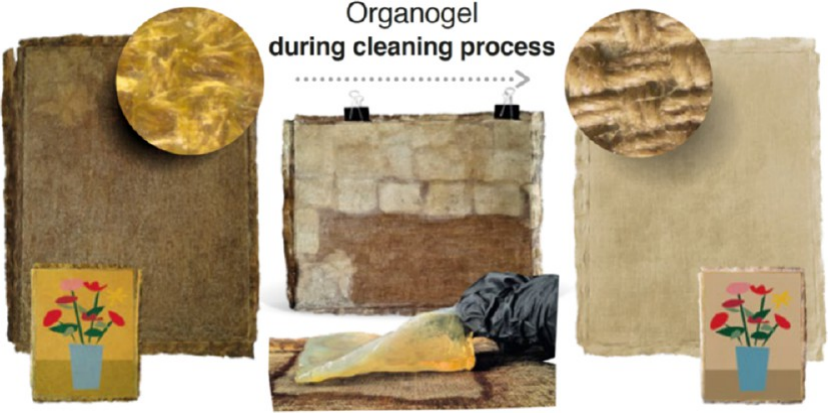

We describe a new, safe, and effective method for removing wax resin adhesive from the canvases of paintings conserved by the once widely used Dutch Method, which involved attaching a new canvas to the back of a painting using an adhesive made of beeswax and natural resin. First, a low-toxicity cleaning mixture for dissolving the adhesive and removing it from the canvases was developed, and then a nanocomposited organogel was obtained. The ability of the organogel to remove the adhesive from canvases was investigated on the lining of the 1878 painting “Battle of Grunwald” by Jan Matejko, with promising results. Additionally, we found that the organogel can be used several times with no visible loss of cleaning ability. Finally, the effectiveness and safety of the method were confirmed on two oil paintings (one from the National Museum in Warsaw): all the wax resin adhesive was removed and the painting regained its original brightness and vivid colors.

## 1 Introduction

Canvas provides the basic substructure for a painting, acting as the medium for paint layers and thus forming an integral part of easel painting. Such a substructure, known as the support of a painting, most often made of linen canvas, has been commonly used in easel painting since the 16th century. On the reverse side of a painting, the canvas is exposed to the long-term destructive effects of numerous factors, such as air pollution, humidity fluctuations, and the growth of microorganisms, resulting in its physical features being weakened.

Restoration of damaged canvases, especially the method of strengthening and securing the support’s fabric, is a crucial issue in the field of conservation and restoration of works of art. One of the most common methods of securing damaged paintings in Europe was the method of attaching a new canvas to the back of the existing one (called “lining” or “relining” the painting) using an adhesive made of beeswax and natural resin. This approach, also known as the Dutch Method, was popularized at the end of the 19th century by Dutch restorers Johannes Albertus Hesterman and his sons; however, its further development is attributed to the restorer Nicolaas Hopman and his son Willem Antonijo and became very popular. The Dutch Method, enjoying high popularity worldwide, ensured the hydrophobicity of the support, simultaneous strengthening of the old canvas and paint layers, and immunity to microorganisms. The wax resin adhesive was also used for securing paint layers and attaching strengthening strips of canvas, as well as consolidating and creating a moisture barrier on the reverse of wood supports and for transferring the paint layer from wood support to canvas.^1^

The wax resin adhesive usually consists of natural or bleached beeswax and natural resin in various proportions. The traditional recipe (Hopman Jr., Hesterman) contained, by volume, 4 parts beeswax, 3 parts rosin, and 2 parts Venetian turpentine.^2−4^ A wax resin adhesive was also often used in the following proportions—6 parts beeswax, 4 parts dammar gum, and 1 part Venetian turpentine.^5^

However, in the 1970s, the Dutch Method came to be criticized, mainly due to darkening of the painting and color changes in paint layers,^6−8^ but also due to the increased weight of the lined painting, the low adhesive strength, the brittleness of the wax resin adhesive, acidification of the canvas (as a result of the addition of rosin), and as a result, acceleration of the aging process of the canvas by the destruction of cellulose. For these reasons, in accordance with modern knowledge in the field of conservation, it is recommended to remove the old wax resin lining that has ceased to fulfill its protective function and replace it with new lining layers that properly strengthen the canvas. Performing the new lining procedure, however, requires complete removal of the old lining and wax resin adhesive from the reverse of the canvas. It is estimated that around 95% of 17th century paintings in the Netherlands have been restored using the lining method.^9^ Although the method was developed in the Netherlands, it has been spread worldwide, and thus, the problem with wax resin lining is global. For example, the National Museum in Warsaw has many paintings that require urgent replacement of lining layers.

Fortunately, the lining procedure is reversible because the wax resin adhesive has a relatively low melting point (60–65 °C).^10^ Removal (subtraction) of the lining canvas attached to the original with the use of wax resin adhesive is an easy procedure thanks to the poor adhesion of this material to the fabric. It is much more difficult to remove the excess of wax resin adhesive remaining on the reverse of the canvas because the adhesive applied while warm seeps into the whole painting.^11^

Various methods for removing the wax resin lining adhesive are known in the art. One of the methods softens the adhesive and then drains it by heating (e.g., ironing through the Japanese tissue paper) or by using a vacuum or low-pressure table.^12−14^ This procedure is sometimes complemented by physical treatment, e.g., using a scalpel or rubbing with sawdust. An extraction procedure is also used, with a solvent or a mixture of solvents being applied in the form of a compress to the treated canvas. Often, restorers develop their own procedure, combining several methods, e.g., solvent extraction alternating with heating, draining using filter paper, and physical treatment. Unfortunately, multiple heat treatment and a poorly selected solvent are detrimental to the painting, while the physical treatment often destroys old and weakened canvas. Among the known methods of removing the wax resin adhesive from the reverse of the painting, beneficial results can be achieved using the solvent method using trichlorethylene (the so-called TCE), applied on cellulose-based carriers (e.g., lignin or sawdust).^14−16^ The use of other solvents (e.g., petroleum ether, kerosene/odorless Mineral Spirits, and white spirit) shows a low surface efficiency in removing the wax resin adhesive. It should be noted that the organic solvents used so far in the process of removing wax resin adhesive, especially TCE, are harmful to health. TCE has strong narcotic,^17^ carcinogenic,^18^ and mutagenic^19^ effects; therefore, its use is not recommended. TCE is included in the list of chemicals for which occupational exposure limits have been established.^20^ TCE should only be used with proper ventilation, which is difficult to ensure when working with large-format paintings and when working in the field. Also, TCE has a low boiling point; thus, it evaporates quickly at room temperature, which increases solvent consumption, and the extraction procedure needs multiple repetitions, which causes a risk to the restorer. However, no effective method of removing wax resin adhesive has been developed thus far whose effectiveness would be comparable to that of TCE. There are also examples of using other organic solvents and mixtures of organic solvents (including turpentine, toluene, xylene, petroleum ether, kerosene, white spirit, trichlorethylene, benzene, dichloromethane, dichloroethylene, and ethanol), which are applied to the reverse of the painting in the form of lignin, cellulose, sawdust, and gel compresses (e.g., Klucel M and Tixogel), and then removed gently or rubbed off.^21^ The tests were carried out on the prepared samples of wax resin adhesive of various compositions (wax, dammar gum, mastic, elemi resin, Venetian turpentine, and rosin), as well as on a real painting, where all use of solvents was complemented by heating and extracting the wax resin material using a low-pressure table. It has been shown that gels soaked with solvents are more effective in removing wax resin adhesive than the solvents themselves or the use of the solvents together with lignin and sawdust.^22^ Unfortunately, this method is not fully effective because after extraction, it was observed that unextracted wax resin adhesive remained in the hollows between the weaves of the canvas, together with a white powder which is the residue left after the evaporation of the gels. Moreover, it has been noticed that extraction using these kinds of gels is not fully controlled in terms of the amount of solvent used and the depth of its penetration. Various attempts to use gels soaked with mixtures of solvents for extracting wax resin adhesive are known, often complemented by the use of a low-pressure table. The use of soft gel carriers usually gives better results than the use of solvents alone, but the undoubted disadvantage of these methods is the contamination of the reverse of the original canvas with remains or fragments of gels used.^21^

The polymeric gels can be defined as cross-linked polymer networks filled with a solvent. A polymer network filled with water gel is called a hydrogel and in the case of organic solvent—organogel. The fluid content in the gels is usually very high and often exceeds 90%; nevertheless, these materials exhibit properties of both liquids and solids. At the macroscopic scale, the gels behave as solid bodies. The tridimensional net is responsible for preserving their actual shape, storing the mechanical energy, and participates in all deformation processes.^23^ In parallel, at the microscale, the gels exhibit liquid properties—diffusional transport of small molecules and ions takes place in them.^24,25^ Such hydrogel properties as absorption of a large amount of solvents, a three-dimensional network that has specific mechanical properties, thermal and chemical resistance, flexibility, and sorption of heavy metal ions and organic compounds are behind the wide use of the gels in many fields.^26−29^ From the perspective of conservation of artifacts, gels are very promising in removing dirt, protective layers, or fixing damage accumulated over centuries.^30^ Various gels, nanomaterials, and composites of gels with nanomaterials have successfully been applied in the field of art conservation for several decades due to their ability to entrap various solvents, their softness, and their ability to absorb and entrap impurities.^31−36^ In art conservation, the porous structure and the ability to absorb the solvent in a polymeric network allow it to be used as a dirt removal material or in consolidation and preservation of stone heritage, as shown by Kim’s group.^30^ Baglioni and co-workers show that semi-interpenetrating poly(2-hydroxyethyl methacrylate)/poly(vinylpyrrolidone) networks have high cleaning capacity for water-sensitive watercolor paintings.^37^ The same group present a polyacrylamide hydrogel-microemulsion system that efficiently removes synthetic adhesives from lined canvas.^38^ The organogels are a narrower group of materials used in art conservation than hydrogels. One of the examples is the organogel based on poly(methyl methacrylate) filled with one of the organic solvents [methyl ethyl ketone (MEK), cyclohexanone (cyclo), ethyl acetate (EA), or butyl acetate (BA)] used for removing old varnish layers for easel painting.^39^ In the literature, the organogels were also successfully used for removing the paraffin from paper work of art,^40^ bio-based organogel with γ-valerolactone (GVL) as an organic solvent was used to clean water-sensitive works of art,^41,42^ and also the reinforced organogel with a γ-valerolactone (GVL) as an organic solvent was used for removing varnishes.^43^

Unfortunately, the existing methods of removing wax resin lining adhesive from the support are insufficient for conservation purposes. The classical chemical method using TCE is effective but poses risks to the health of the restorer. On the other hand, other methods, including methods using gel carriers, are significantly safer, but without the need for heating, draining, or mechanical removal of residues arising during the extraction process, they do not provide sufficient efficacy. The selection of a gel carrier for this purpose is still challenging. Organogels do not usually absorb large amounts of solvent and maintain appropriate mechanical properties.

The aim of this study was to provide an effective and safe, for both conservers and works of art, method for removing wax resin lining adhesive from the reverse of paintings. We focused our attention on researching a low-toxic solvent mixture whose effectiveness could be comparable to the currently used solvents with high harm potential to humans, such as TCE. The second important goal of this study was to obtain a gel carrier which created the organogel with the developed solvent mixture, with the properties desired for the safety of conservation of works of art. The work reported herein is the subject of patent application no. PCT/PL2021/000044.

## 2 Materials and Methods

### 2.1 Materials

The reagents for the synthesis of gels: *N*-isopropylacrylamide (NIPA) from Fluorochem Ltd., *N*,*N*′-methylenebisacrylamide (BIS) from Merck, and Laponite XLS synthetic hectorite nanoclay (Lap) (92.32 wt % Mg_5.34_Li_0.66_Si_8_O_20_(OH)_4_Na_0.66_ and 7.68 wt % of Na_4_P_2_O_7_) were kindly provided by BYK-Chemie GmbH, and sodium persulfate (NaPS) and *N*,*N*,*N*′,*N*′-tetramethylethylene diamine (TEMED) were purchased from Aldrich. Acetone, isopropanol, isooctane, ethanol, methanol, hexane, and cyclohexanone were obtained from POCh (Poland). All solvents were pure P.A. All chemicals were used as received except for NIPA, which was recrystallized from a toluene–hexane mixture (3:7 v/v). Synthesis was prepared using high-purity water obtained from a Milli-Q Plus/Millipore purification system (water conductivity: 0.05 μS·cm^–1^).

### 2.2 Preparation pNIPA-LAP Organogel

A pNIPA-LAP hydrogel was synthesized by free-radical solution polymerization. First, Laponite (60 mg/mL) was dispersed in deionized water and ultrasonicated for 30 min. Then, the NIPA monomer was added to the solution (the NIPA concentration was 1 M). The solution was stirred and deoxygenated for 1 h in an ice bath, and then a catalyst (TEMED, 5 μL/mL) was added. After 15 min of stirring, the initiator (NaPS, 2 mM) was added, and the pre-gel solution was transferred to a vessel. Polymerization was performed at 20 °C for 24 h. Sheets of hydrogels with various dimensions were obtained. After polymerization, the prepared hydrogels were soaked with deionized water several times to remove residues. After cleaning, the hydrogels were dried at 30 °C for several days. The drying process allowed the water solvent to be exchanged for a mixture of organic solvents POA (35% v/v isopropanol, 45% v/v isooctane, and 20% v/v acetone, detailed description is given in Section 3.1). The gel was left for 2 days to swell in the mixture. In this way, a nanocomposite organogel was obtained. After this step, the organogels were cut into appropriately sized pieces. The NIPA-LAP organogel is pale white/opalescent in color, but it does not lose its transparency.

### 2.3 Methods

#### 2.3.1 Swelling Ratio Measurements

For swelling ratio determination, hydro- and organogels were cut into regular shapes, dried, weighted, and then immersed in a solvent for 2 days to obtain the equilibrium swelling ratio, after which the pieces of gel were removed from the solution, the excess of solvent was wiped off, and the gels were immediately weighed on a Radwag XA 52/2X balance.

The solvent content was calculated using eq 1
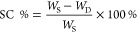
1where *W*_S_ is the mass of the swollen gel and *W*_D_ is the mass of the dry gel.

#### 2.3.2 Scanning Electron Microscopy Investigation

Scanning electron microscopy (SEM) (Zeiss Merlin Field emission) was used for the examination of hydrogel morphology. To capture the pore size of the swollen hydrogel, samples were freeze-dried. Thus, the first samples were frozen in liquid nitrogen to maintain the porous structure of the gels and then lyophilized on a Labconco FreeZone Lyophilizer conditions: temperature −82 °C and vacuum 0.03 mbar. Finally, the samples were sputtered with a palladium-rod layer. Due to specific condition of the lyophilization process, the freezing of polymer structure is possible only for hydrogel.

#### 2.3.3 Mechanical Property Measurements—Tensile Tests

The mechanical tests were performed using a Shimadzu universal tensile machine equipped with a 20 N load cell. Tensile tests were performed on gel rectangle samples of 10 × 2 × 15 mm. Compressive tests were performed with gel samples (10 mm diameter and 5 mm length). Measurements were made at room temperature with a crosshead speed of 50 mm·min^–1^ for tensile tests and a constant speed of 5 mm·min^–1^ for compressive tests. Compression was performed up to 95%.

#### 2.3.4 Rheological Investigation

The Anton Paar MCR302 rheometer was used for dynamic shear rheology experiments using a set of 15 mm diameter sandblasted parallel plates at a constant temperature of 20 °C. First, dynamic oscillatory amplitude sweep experiments were performed on the hydro- and organogels to determine the limit of the linear viscoelastic region. The dynamic strain sweep (γ) was performed at a constant frequency, ω = 10 rad·s^–1^ in the range from 0.01 to 500%. Therefore, in all the frequency sweep tests, the strain amplitude (γ) was fixed at 1% (within the linear viscoelastic range which was small enough to avoid the nonlinear response and large enough to have a reasonable signal intensity) over a frequency range of 0.01–100 rad·s^–1^. The temperature was controlled using a PolyScience circulating bath. To keep the constant temperature of gel samples and minimize solvents evaporation during rheological measurements a special cap was used.

#### 2.3.5 Canvas Origin Used for the Cleaning Test with the Usage of pNIPA-LAP Organogel

All laboratory experiments were conducted using small samples of the lining canvas from the 1947 lining of the painting “*Battle of Grunwald*” by Jan Matejko (in the collection of the National Museum in Warsaw), dating to 1878.

For tests in conservation laboratory, where comprehensive procedure of restauration was performed, two original oil paintings were selected: “*Zinnias in a Blue Vase*” from 1930, artist unknown, around 1950 restored using the Dutch Method (painting from private collection), and “*Tsarina Catherine*” dated to the 18th century, artist unknown, date of restoration unknown (painting in the collection of the National Museum of Warsaw).

#### 2.3.6 Infrared Spectroscopy Investigations

An Alpha FTIR spectrometer by Bruker equipped with a QuickSnap ATR module with a diamond crystal was used to record the IR spectra of canvases before and after treatment with the mixture of organic solvents.

#### 2.3.7 Gas Chromatography Coupled with Mass Spectrometry Measurements

Tests carried out using gas chromatography coupled with mass spectrometry (GC–MS) were used to determine the influence of the extraction using a cleaning mixture (POA) on the chemical composition of the oil paints. The paints were prepared with linseed oil and two pigments: lead white and Cyprus Umber. The fresh paints were aged at 80 °C using UV radiation (310 W UVBHAND 250 GS, Honle UV technology) for 24 h. Then, from each paint, two samples weighing ca. 2 mg were collected, one of which was exposed to a 30 min POA treatment and the other was used as a reference sample. After treatment with the POA mixture, the samples were centrifuged, and then the solvent was separated from the sediment. The residual solvent was removed by evaporation with a gentle stream of nitrogen at 60 °C. Then, the samples were prepared for GC–MS analysis, with 200 μL of methanol/toluene mixture (1:2, v/v) and 60 μL of a 1% solution of *m*-trifluoromethylphenyl trimethylammonium hydroxide (TFTMAH). The sample was initially placed in the ultrasonic field for approx. 30 s and then heated for 60 min at 60 °C. After the end of the transesterification reaction, the solution was centrifuged from the insoluble residue and analyzed using GC–MS. The analysis was carried out using a GC–MS-QP2010 Ultragas chromatograph (Shimadzu) with a single quadrupole QP-5000 mass spectrometer (Shimadzu). The analytes were separated with a HP-5MS-plus (Agilent) capillary column: 30 m × 0.25 mm, 0.25 μm of the stationary phase. The flow of carrier gas (He) was 9.5 mL/min, and the split ratio was 10. The injector, ion source, and the mass spectrometer transfer line temperatures were kept at 300 °C, and the following temperature program was used: initially, 50 °C hold for 1 min and then a linear increase at the rate of 10 °C/min to 320 °C. The column oven temperature was 320 °C for 12 min, and the analysis was completed in 38 min. Electron ionization was used to ionize the analytes eluting from the column and the mass spectrometer operated in the scan mode in the mass range of 35–500 *m*/*z*; the solvent cut was 10 min. The GC–MS solution 2.53 (Shimadzu) program was used for data acquisition and processing.

## 3 Results and Discussion

### 3.1 Mixtures of Organic Solvents for Extraction of a Lining Adhesive

**Figure 1:**
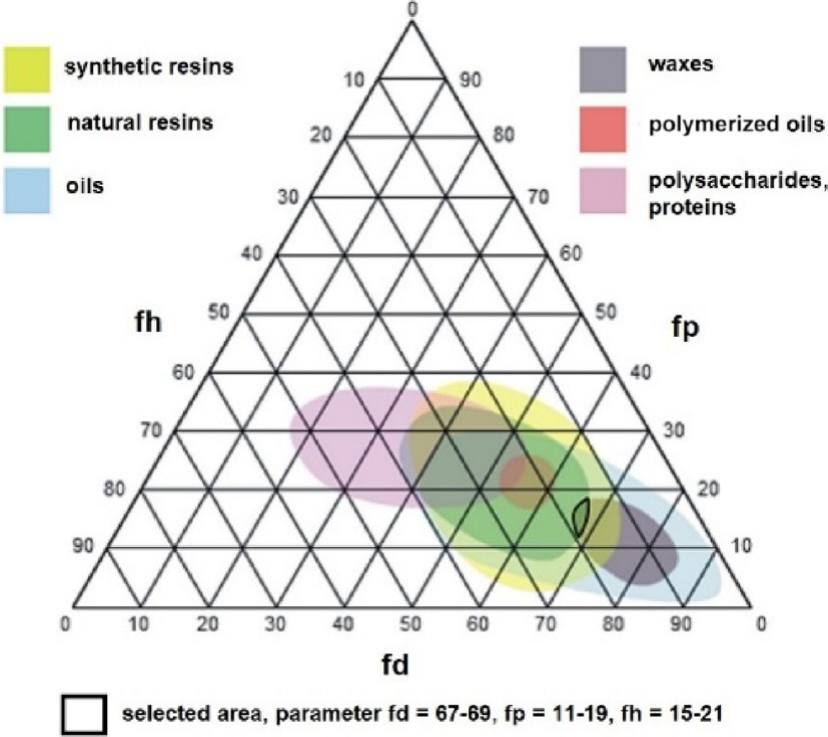
Teas’ diagram for paint components. The black outlined area shows the overlapping parameters of natural resins, waxes, and synthetic resins on the basis of which the composition of the cleaning mixture was selected.

The first step of the investigations involved seeking out and testing novel cleaning mixtures for the extraction of lining adhesive. For this purpose, the methodology developed by Teas in 1968.^44−46^ was employed, which assumes that each solvent is characterized by three parameters determined mathematically on the basis of Hansen parameters. Teas’ parameters define dispersion force (fd), polar force (fp), and hydrogen bonding force (fh),^47,48^ the sum of which is constant and equals 100 (fd + fp + fh = 100).^44,45,49^ These parameters determine the abilities of a solvent or mixture of solvents to dissolve/remove various materials which can be present in paintings. Figure 1 shows an approximated area, defined by Teas’ parameters, appropriate for the removal of these substances. The analysis of the graph allowed the parameters of the cleaning mixtures in the range safe for paintings to be determined, so that their properties cover the area common to waxes and resins, and are beyond the area of the polymerized oils which can be found in paintings, and the polysaccharides and proteins present in canvases. The most interesting area in Teas’ graph is marked by the black outline and cover parameter values: dispersion force fd = 67–69, polar force fp = 11–19, and hydrogen bonding force fh = 15–21 (see Figure 1). Although the Teas’ methodology is not a perfect tool,^50^ it allows the properties of novel solvent mixtures to be predicted to some extent.^51,52^ Teas’ method was used to develop three-component mixtures capable of dissolving waxes and resins simultaneously. Commonly used available interactive applications were used: “Modular Cleaning Program”^53^ and “TriSolv”.^54^ We found that non-toxic cleaning mixtures exhibit parameters similar to the parameters of trichloroethylene (TCE) (68-12-20) and other currently used toxic solvents, e.g., chloroform (67-12-21), dibutyl ketone (67-16-17), and 1,2-dichloroethane (67-19-14).^48^ Potential three-component mixtures were preselected, the use of which would ensure effective removal of the wax resin adhesive and an increase in safety for conservators. The components of the mixtures were selected from cheap, low-toxicity organic solvents commonly used in conservation and chemical laboratories (e.g., ethanol, isopropanol, hexane, isooctane, acetone, petroleum ether, and white spirit).^55^ Toxic solvents (e.g., xylene and toluene) were excluded, even if their effectiveness in removing the wax resin adhesive was known. The search was conducted with the aim of maximizing the affinity of the cleaning mixture to the components of the wax resin adhesive, containing hydrocarbons (including fatty acids, alcohols, and esters) with mainly hydroxyl, carbonyl, and carboxyl groups. Three-component cleaning mixtures containing alcohol, hydrocarbon, and ketone components (AHK) were selected for further research.

Two three-component mixtures containing components from the classes defined above were preselected: ethanol/isooctane/acetone (EOA) and isopropanol/isooctane/acetone (POA), whose compositions were designed to meet the requirements of Teas’ parameters: EOA—ethanol: 30–36%, isooctane: 42–48%, acetone: 15–22%; POA—isopropanol: 32–40%, isooctane: 41–46%, and acetone: 17–24%. Then, an experimental verification of the earlier predictions was performed in order to determine the optimal composition of these ternary mixtures. Several compositions of ternary mixtures corresponding to the boundary and intermediate values of Teas’ parameters were selected and tested in the removal of wax resin adhesive.

Comparative tests of extraction of wax resin lining adhesive were carried out according to the procedures traditionally used in the conservation of paintings to determine the effectiveness of EOA and POA mixtures in comparison to classically used solvents. All experiments were conducted using small samples of the lining canvas from the 1947 lining of the painting “Battle of Grunwald” by Jan Matejko (from the National Museum in Warsaw), dating to 1878. A four-layer sheet of lignin moistened with a solvent was placed on the canvas and covered with Melinex foil. The procedure was carried out for 60 min. Wood sawdust was not used since mechanical removal of the residue from the hollows between the weaves of the canvas would damage the weak fibers of the fabric. Effective extraction of EOA and POA was compared with TCE. After the use of these solvents, a characteristic lignin color was observed, originating from the dissolved wax resin adhesive, and the structure of the linen fiber was uncovered, although the wax resin adhesive remained in its deeper parts and in clusters on the surface, in the areas where it was previously present in a thicker layer. The experiment confirmed the effectiveness of EOA and POA mixtures in removing the wax resin lining adhesive, which was comparable with the effectiveness of the classically used toxic TCE. Slightly better results were obtained for the POA mixture, and for further experiments, this mixture (isopropanol 35%, isooctane 45%, and acetone 20%; Teas parameters: 68.8–12.0–19.3) was selected.

### 3.2 Structure and Mechanical Properties of pNIPA-LAP Gels

**Figure 2:**
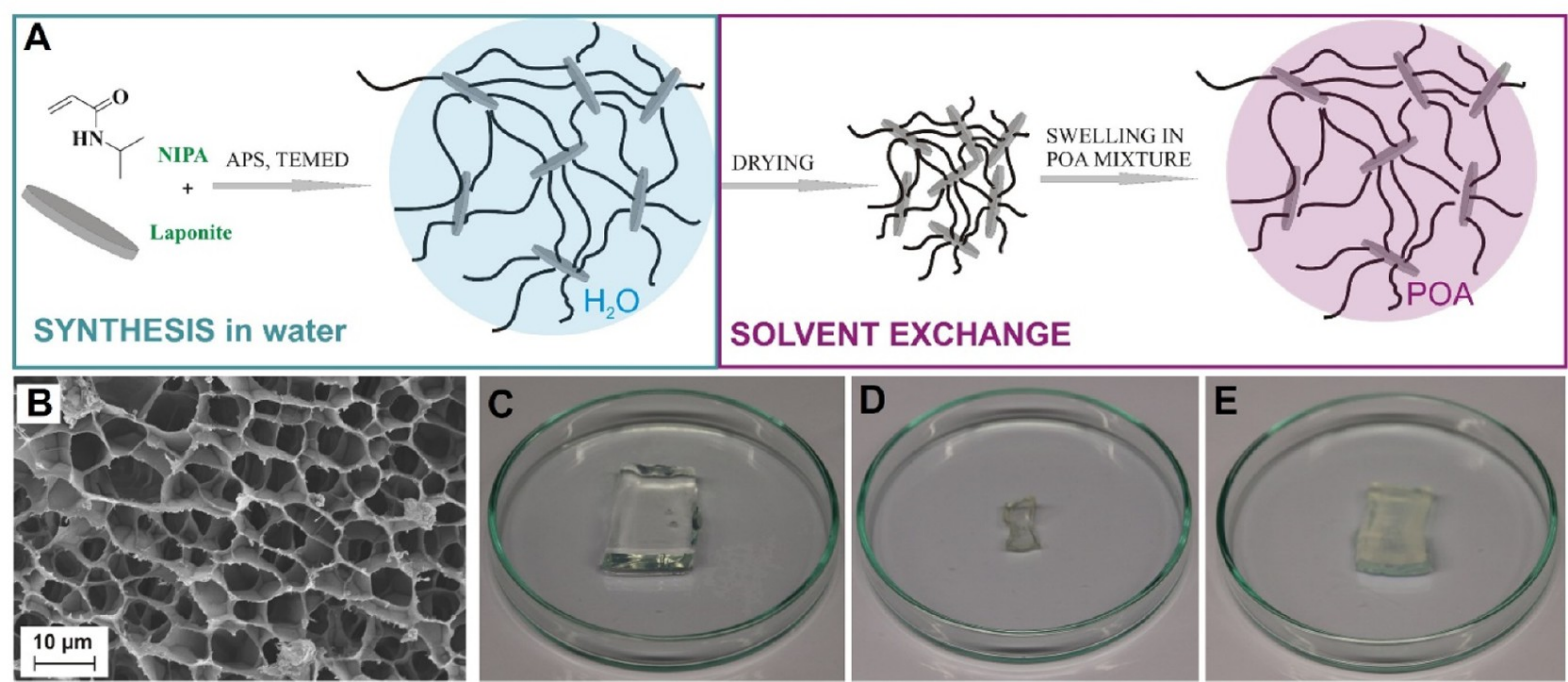
(A) Scheme of the synthesis of pNIPA-LAP hydrogel and preparation of pNIPA-LAP-AHK organogel. (B) SEM images of the freeze-dried hydrogel pNIPA-LAP. Photos of the sample at each step of preparation: (C) hydrogel pNIPA-LAP, (D) dried pNIPA-LAP, and (E) organogel pNIPA-LAP-AHK.

The next challenging step involved finding the appropriate carrier for the POA mixtures. From the point of view of the properties of the system desired for extracting the wax resin lining adhesive, i.e., the capability to absorb large amounts of solvent mixtures, limiting the possibility of leakage of the solvent mixture, high elasticity, cohesiveness, and mechanical strength, polymeric gels were selected. For this purpose, polymer gels based on poly(*N*-isopropylacrylamide) (pNIPA) that have specific amphiphilic properties were chosen. Hydrogels based on poly(*N*-isopropylacrylamide) cross-linked with *N*,*N′*-methylenebisacrylamide (BIS) were synthesized according to the procedure described elsewhere^56^ and were then purified and dried. The dried polymers obtained were then swelled in the POA mixture until it reach equilibrium swelling ratio. Additionally, it was observed that the cross-linked polymer did not swell in TCE. Then, tests were carried out to extract an old wax resin lining adhesive from the canvas using the pNIPA-POA organogel. The effect of the extraction was very good; after less than 1 h, the canvas showed brightening and exposed fabric fibers. Observing the canvas under a microscope confirmed the effectiveness of the extraction of wax resin adhesive, including from the hollows between the weaves of the canvas. Unfortunately, the mechanical strength of the organogels based on the pNIPA matrix was insufficient, and the gel showed a tendency to break. To improve the mechanical properties, the cross-linker BIS was exchanged for a nanostructured Laponite XLS (LAP). This strategy was proposed by Haraguchi for hydrogels.^57^ The scheme of synthesizing pNIPA-LAP hydrogels and preparing the pNIPA-LAP organogel with relevant photos of real samples are presented in Figure 2. As can be seen, the hydrogel has a highly porous structure (Figure 2B), and the solvent content seems to be high (Figure 2C–E). The solvent content in the hydro- and organogel was determined by measuring the weight of gel sheets in water and the POA mixture at room temperature. For the hydrogel, the water content was ca. 95% and the organogel contained ca. 87% percent organic solvent mixtures of POA.

**Figure 3:**
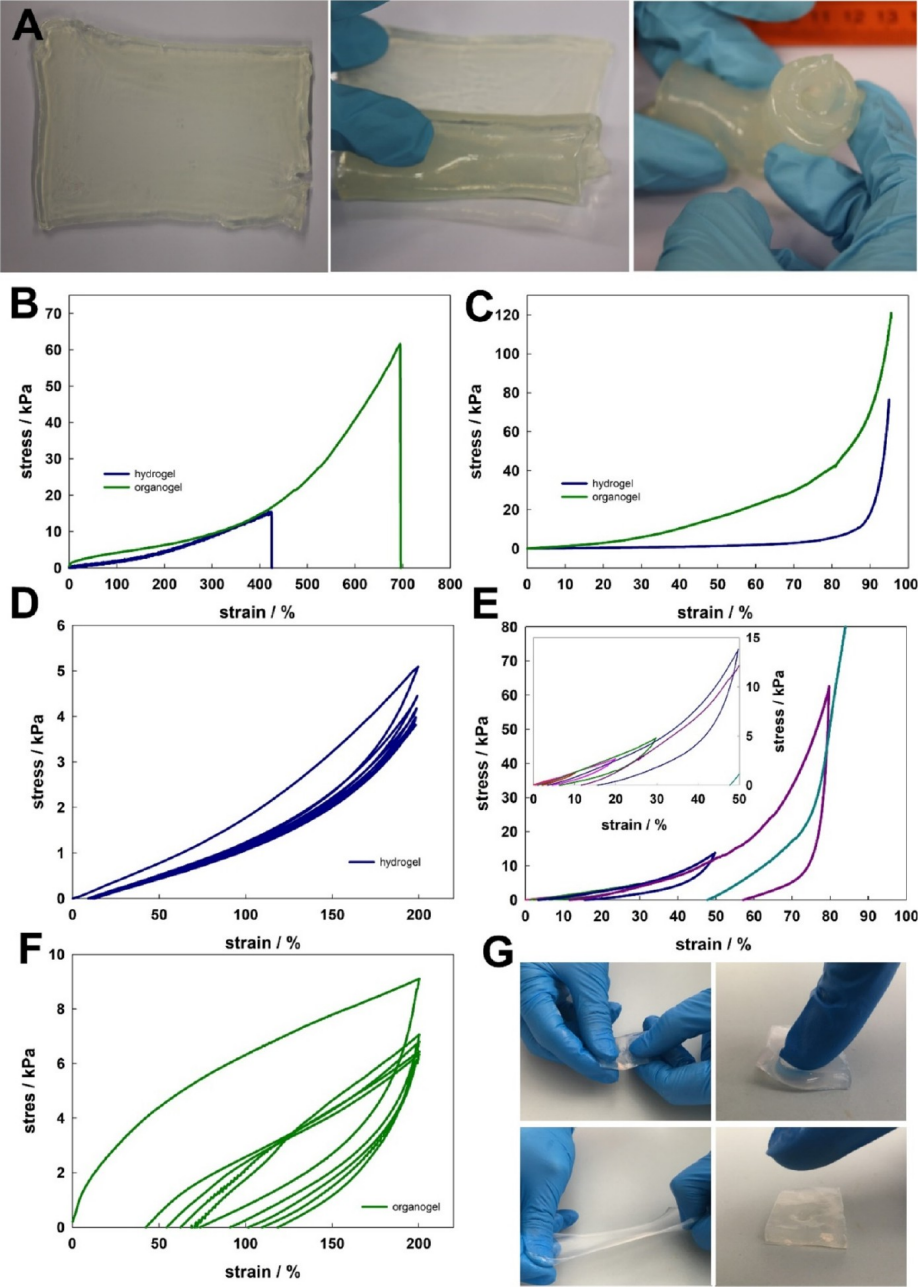
(A) Photos of rolling of NIPA-LAP organogel. (B) Elongation stress–strain curve for NIPA-LAP organogel (green line) and hydrogel (blue line). (C) Compression stress–strain curve for NIPA-LAP organogel (green line) and hydrogel (blue line). (D) Loading–unloading elongation curves for NIPA-LAP hydrogel (five cycles). (E) Loading–unloading compression curves for NIPA-LAP organogel (five cycles first 10%, second 20%, third 30%, fourth 50%, fifth 80%, and last compression stopped at 82%). (F) Loading–unloading elongation curves for NIPA-LAP organogel (five cycles). (G) Images show stretching of NIPA-LAP organogel (left column) and compression of NIPA-LAP organogel (right column).

Next, nanocomposite organogels were subjected to mechanical tests using a tensile machine, and organoleptic evaluation was also performed. The material showed no tendency toward sticking together, and it underwent multiple rolling into a roll (Figure 3A), which is important for practical reasons as it provides convenient storage, transport, and other activities prior to use. Results of stretching and compressive tests for the organogel and hydrogel are presented in Figure 3B,C, respectively. Tensile tests showed that the organogel can be stretched much more than the hydrogel without mechanical damage. The maximal compression stress was also higher for the organogel, see Figure 3C. Characteristic mechanical parameters, e.g., elongation at break, max. stress, toughness, and max. stress compression (in 95% of compression), were significant higher for the organogel, as follows (organogel/hydrogel): 700/420 (%), 61.5/15.0 (kPa), 127/25 (kJ/m^3^), and 120/75 (kPa), respectively. Next, the cyclic compression test with increasing compression percentage was performed (Figure 3E). 10, 20, 30, 50, and 80% of compression with 10 s quiet time between consecutive cycles were selected. The last test was terminated at 82% of compression. It can be noted that up to 30% compression rather viscoelastic deformation was observed with good organogel recovery and returning to the initial height/shape. For higher compression (50 and 80%), the hysteresis was relatively large, the recovery was very poor, and the material did not return to its original height/shape at rest, suggesting that plastic deformation took place. Cyclic experiments were also conducted for elongation at 200% strain. In the case of the hydrogel (Figure 3D), a small hysteresis loop was observed in the load–unload curves in the first cycle, and it decreased significantly in the other four cycles, indicating energy dissipation in the first cycle and elastic behavior in the others. For the organogel (Figure 3F), the significantly bigger hysteresis loops were observed, indicating that energy was dissipated during the loading process, but the curves did not return to the origin points, indicating the plastic deformation of the organogel. The observed differences in organogel vs hydrogel behavior could be caused by the rearrangement of the polymer network during the preparation of the organogel. The obtained hydrogel was perfectly transparent, and after drying and re-swelling in the mixture of solvents, the resulting organogel became opaque. The inhomogeneity of the polymer network may be due to the irregular distribution of cross-linking points (agglomerates of clay nanoplatelets could be formed) and/or the coiled structure adopted by pNIPA chains in a less favorable environment.^58,59^ It should also be noted here that the results obtained for the organogel were affected due to the evaporation of the solvent during the long-term test—evaporation is faster for the organogel than for the hydrogel.

As can be seen in Figure 3B,C, the pNIPA-LAP organogel has better mechanical properties for the purposes presented here than the pNIPA-LAP hydrogel. The organogel underwent deformation, and after the force ceased, the gel retained its original shape (Figure 3G, left column); importantly, the release of a liquid mixture of solvents from the pNIPA-LAP organogel under the applied forces was also not observed, which is a significant advantage over classic carriers (e.g., sawdust and lignin).

**Figure 4:**
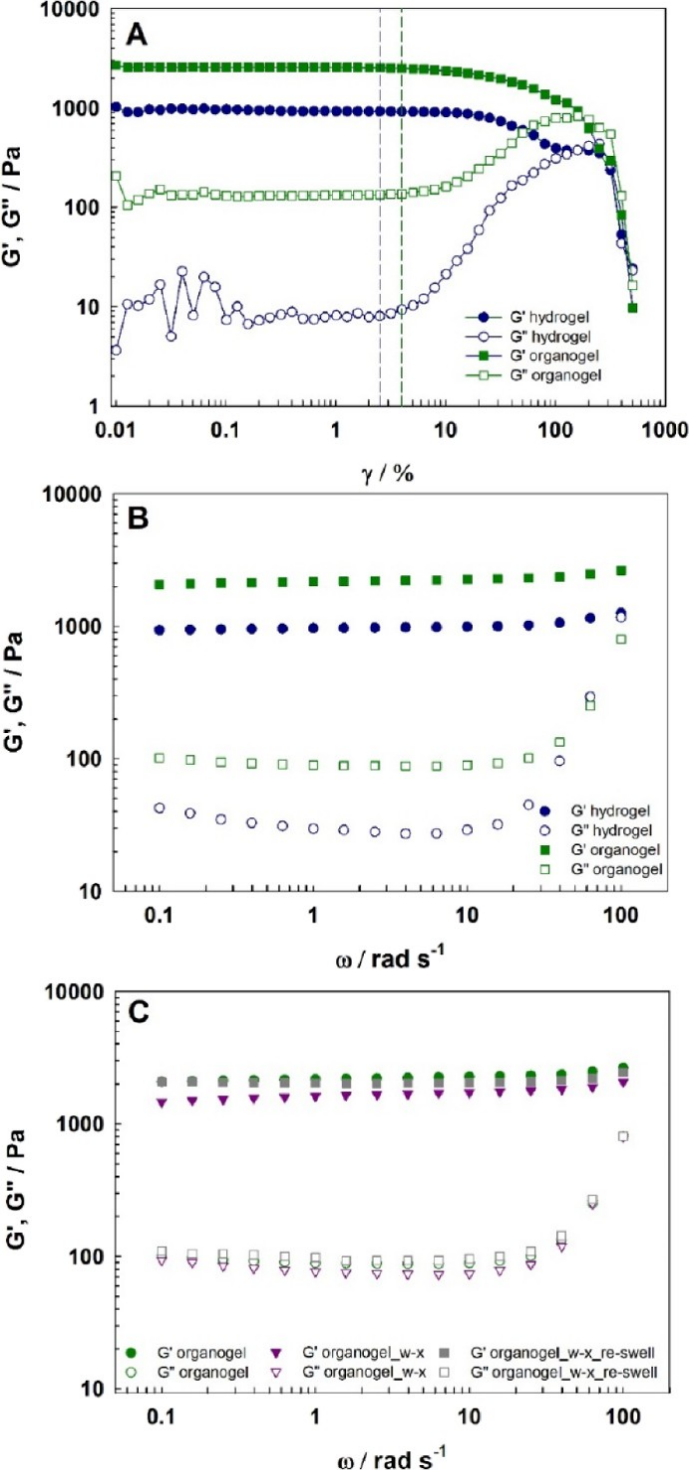
(A) Storage (*G*′, solid symbols) and loss modulus (*G*″, hollow symbols) as a function of shear strain for hydrogel (blue circles) and organogel (green squares). (B) Storage (*G*′, solid symbols) and loss modulus (*G*″, hollow symbols) as a function angular frequency (at γ = 1%) for hydrogel (blue circles) and organogel (green squares). (C) Storage (*G*′, solid symbols) and loss modulus (*G*″, hollow symbols) as a function of angular frequency (at γ = 1%) for organogel: before application on canvas (green circles), after using for wax resin removal and washed (purple triangles), and after drying and re-swelling in POA mixture (gray squares).

Dynamic mechanical analysis was performed for further mechanical and structure investigation of the organogel. The design of the measuring cell (equipped with a hood and grooves with the solvent mixture) allowed the evaporation of the solvent mixture to be limited for the duration of the test. First, amplitude sweep shear strain (γ) measurements were performed to determine the liner viscoelastic region (LVR) for both hydro- and organogels. Figure 4A shows the storage modulus (*G*′) and loss modulus (*G*″) as a function of shear strain for a fixed frequency 10 rad/s. The organogel was characterized by a slightly wider LVR viscoelastic region than the hydrogel, and the critical strain γ_c_ values were equal to 2.5 and 4% for the hydro- and organogel, respectively, and were marked with the dashed lines in Figure 4A. In the LVR region, *G*′ and *G*″ are independent of shear strain. In this region, the storage modulus is significantly higher than the loss modulus *G*″, which indicates that both gels were in a solid-like state. Figure 4B presents *G*′ and *G*″ at different angular frequencies for the hydro- and organogels, with a constant sweep amplitude of γ = 1% selected based on Figure 4A from the LVR. Storage modulus values were more than 2 times higher for the organogel, *G*′ = 2260 Pa, than for the hydrogel, *G*′ = 990 Pa, indicating that the organogel had a more rigid structure (being stiffer), while the hydrogel was a softer gel material. Nevertheless, for both materials, *G*′ was almost independent of frequency, while the loss modulus *G*″ indicating the viscous nature of the material increased with increasing frequency. For ω = 10 rad/s *G*″ equaled 90 Pa for the organogel, 28 Pa for the hydrogel. For higher frequencies, the timescales of deformation were short, and the viscous response heavily increased due to the undisturbed movement of polymer chains during the timescale of applied shear stress (the clay cross-linked polymer network is characterized by long mobile polymer chains compared with conventional, chemically cross-linked hydrogels),^60^ and the loss modulus approached the storage modulus. Additional measurements were taken to evaluate the changes in the organogel during the wax and resin removal application and during drying. Figure 4C shows the storage modulus and loss modulus upon frequency changes for one piece of organogel during processing in the following stages: (a) fresh organogel; (b) organogel after application on a wax resin covered canvas for 20 min and recovered in a fresh batch of the POA mixture (organogel_w-x), and (c) after air-drying and re-swelling in the POA mixture (organogel_w-x_re-swell). As can be seen, there is no significant change in the frequency dependence of *G*′ and *G*″ moduli, and *G*′ and *G*″ values for organogel_w-x and organogel_w-x_re-swell are not significantly different from those of fresh organogel. This result is consistent with data for nanocomposite hydrogel, where mechanical properties change after first drying (in our study, the first drying was after synthesis) and remain constant after further drying–swelling cycles.^61^

### 3.3 Cleaning of Canvas with the NIPA-LAP Organogel and Regeneration of the Organogel Sorbent

**Figure 5:**
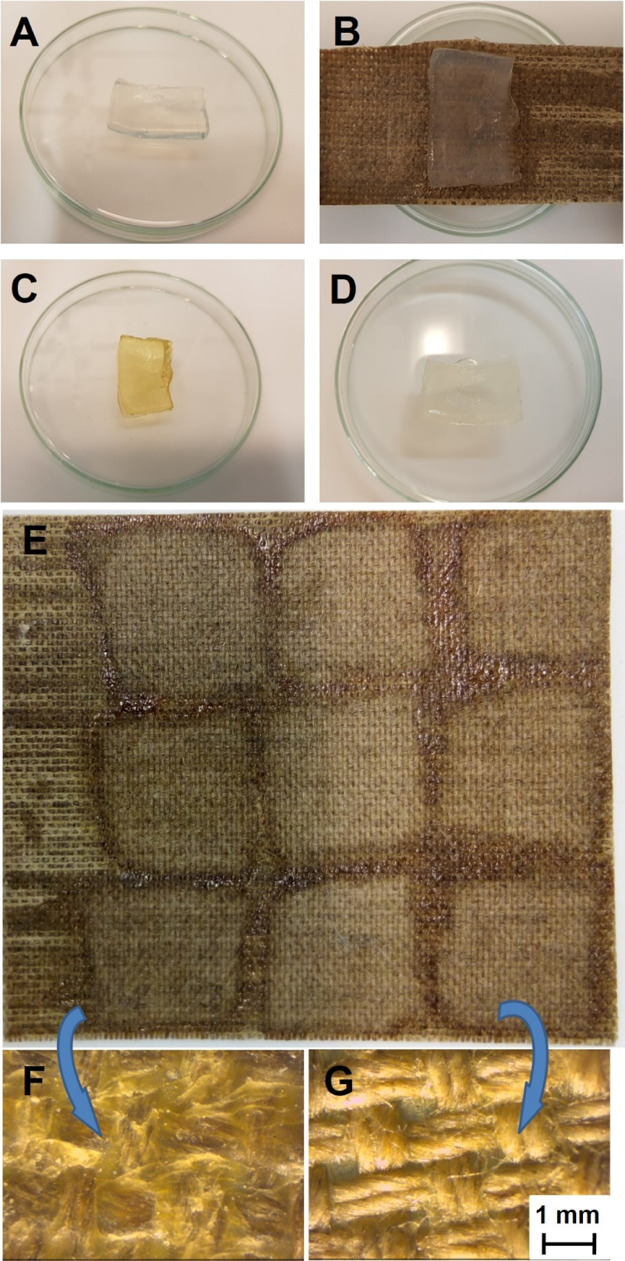
Photos of pNIPA-LAP organogel piece before cleaning (A), during cleaning (B), after cleaning (C), and after recovery (D). Canvas after nine steps of cleaning with pNIPA-LAP organogel with one gel piece used nine times with a recovery step between cleaning (E), and optical microscopic photo of canvas before (F) and after the cleaning process (G).

Next, the ability of the pNIPA-LAP organogel to remove the wax resin adhesive from the canvas was investigated. For this purpose, the organogel was cut into samples 3 cm × 2 cm × 0.3 cm and applied for 1 h on the canvas. To prevent evaporation of the solvent, the organogel was covered with thin polyethylene foil. After the cleaning step, the gel was immersed in a fresh portion of POA mixture to remove the absorbed doublage mass. As shown in Figure 5A–D, the wax resin was removed by the organogel from the canvas, and the gel turned orange as a result of the dissolution and sorption of the wax resin doublage layer. The wax resin adsorbed in the pNIPA-LAP organogel can easily be removed by washing in a fresh solvent. The effect of washing can be clearly seen in Figure 5A,D, where the photo of pNIPA-LAP before and after regeneration can be compared. Repeatability tests of the extraction process were carried out by cleaning nine different areas of the canvas with the same piece of pNIPA-LAP organogel for 1 h, with regeneration after each cleaning (Figure 5E). As can be seen, regeneration of the gel can be carried out numerous times without noticeable changes in the cleaning ability. In the places where the gel is in direct contact with the fabric, visible brightenings appeared, proving the removal of the wax resin adhesive, which was confirmed by microscopic examination (Figure 5F,G). Effective removal of wax resin adhesive from the reverse of painting was comparable with TCE.

**Figure 6:**
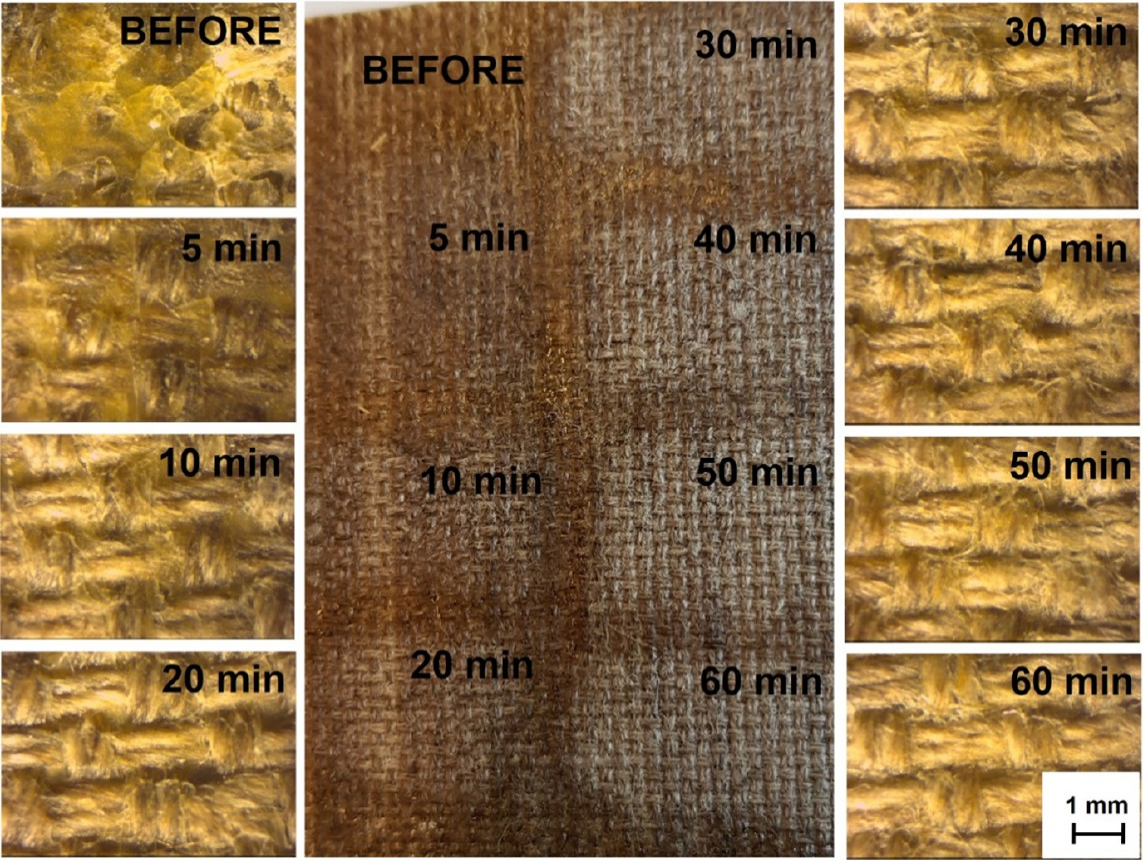
Photos and optical microscopic images of canvas before the cleaning process and after the cleaning process lasting, respectively, 5, 10, 20, 30, 40, 50, and 60 min.

To estimate the optimal time for the cleaning step (the contact of organogels with the canvas), the extraction time was variable from 5 min to 1 h. A series of seven extractions were carried out. After each extraction, the piece of gel was regenerated for 1 h in a mixture of organic solvents. A photo of the cleaned canvas and microscopic images are shown in Figure 6. It was found that after 30 min, satisfactory removal of the wax resin adhesive even from between the fibers of the canvas was observed.

### 3.4 Test of the Influence of the Solvent Mixture on the Canvas and the Painting

The potential unwanted influence of the pNIPA-LAP organogel on paintings is a very important issue. Because wax resin adhesives permeate throughout the painting,^46^ the neutrality of the organic mixture in terms of the paint layers, including canvas, pigments, and binder, were investigated.

**Figure 7:**
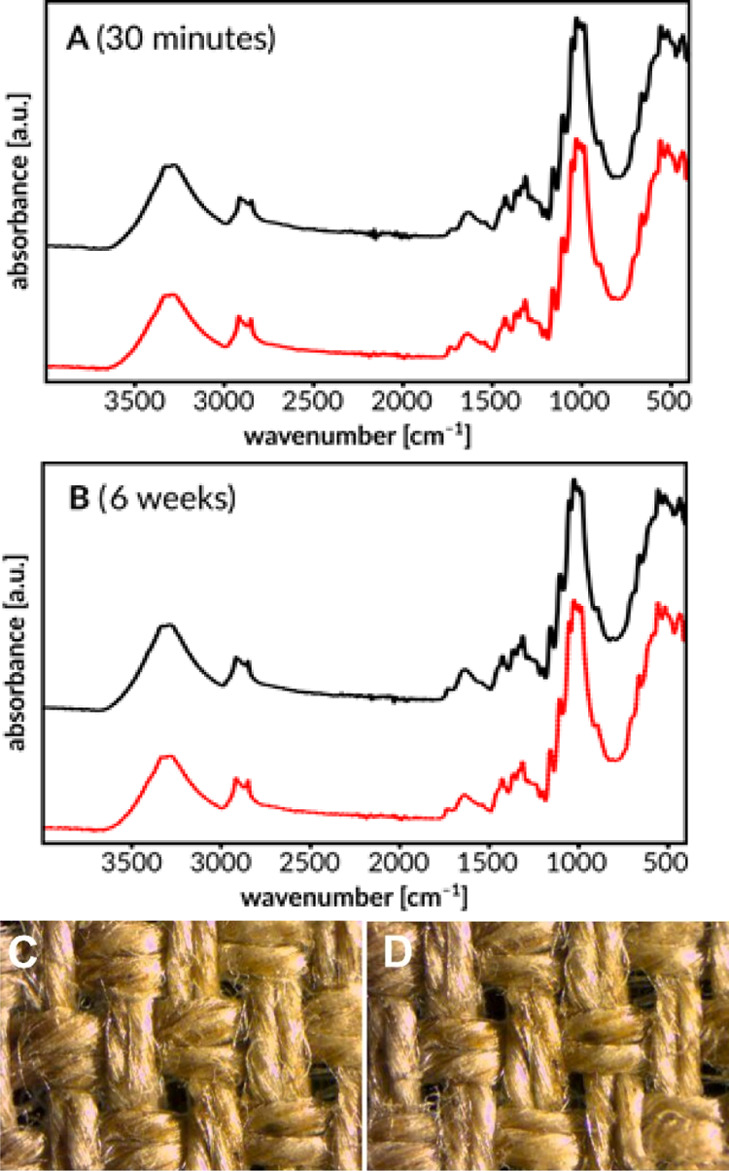
FTIR spectrum of the canvas soaked in the POA mixture for 30 min (A) and 6 weeks (B). (Reference spectra recorded for untreated canvas marked with red lines), optical microscopic images of canvas before (C) and after treatment with the POA mixture for periods of 6 weeks (D).

The resistance of the decatized linen canvas to the treatment with the organic mixture was verified by comparative infrared spectroscopy measurements. Samples of the canvas were treated with the POA mixture for periods of 30 min and 6 weeks. Subsequently, the samples of the canvas tested were subjected to FTIR examination and compared with the spectrum of pure linen. The results are shown in Figure 7. The spectra of the canvas treated with the cleaning mixtures (black lines), even after 6 weeks, do not show any significant differences from the spectrum of the clean canvas (red lines). Thus, it can be concluded that the mixture of organic solvents used does not chemically interact with the canvas. Also, investigations of the canvas by optical microscopy have not shown any noticeable differences between the treated and untreated canvases.

**Figure 8:**
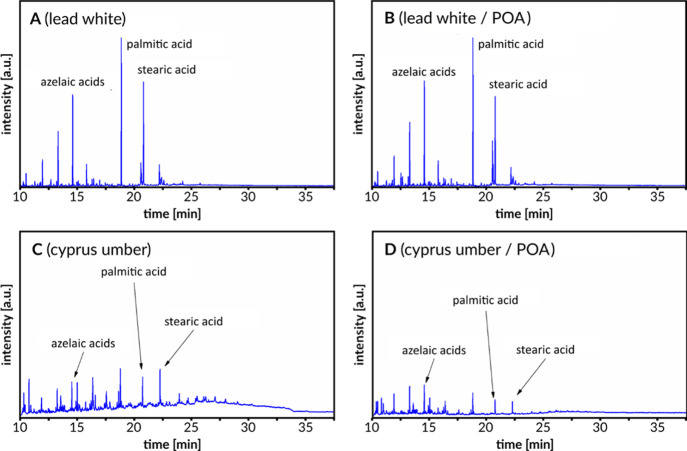
Chromatograms obtained for the aged oil paints before (A,C) and after treatment with POA mixture (B,D).

The influence of the POA mixture on degraded oil paints was also studied. Two oil paints were prepared: one with linseed oil (binder) and lead white (pigment), while the second contained linseed oil and Cyprus Umber. Next, aged paints under accelerated conditions—80 °C and UVB–vis radiation for 24 h—were treated with POA for 30 min. Comparative research using GC–MS was carried out, and the results are presented in Figure 8. Unsaturated fatty acids, in the form of glycerin esters, are the main ingredients of fresh natural oils.^62^ Oil drying and aging are associated with a decrease in the content of unsaturated acids as a result of oxidation of the double bonds of oleic acids and linoleic acid and by polymerization.^63^ As a result of their oxidation, dicarboxylic fatty acids are formed, mainly azelaic acid and suberic acid.^3^ For this reason, dried and old (degraded) oil binders are characterized by high acid content dicarboxylic acids.^3,64,65^ As can be seen in all the chromatograms, the signal from azelaic acid is substantial. This means that the aging process was successful. Components of oil binders which, unlike unsaturated fatty acids, are much less susceptible to degradation are saturated fatty acids: palmitic acid and stearic acid.^3,66,67^ A common method of identifying oil binders is based on the determination of the relative content of azelaic acid to palmitic acid (A/P) and palmitic acid to stearic acid (P/S).^68^ This method was used to evaluate the influence of the POA mixture on degraded oil paints. Relative fatty acid contents were determined from area ratios under characteristic chromatographic peaks. In the case of the lead white paint, the P/S ratios before and after contact with the POA were 1.56 ± 0.10 and 1.48 ± 0.11, respectively. In the same case, the A/P ratios before and after contact with the POA were 0.91 ± 0.14 and 0.85 ± 0.20, respectively. For the Cyprus Umber paint, analogous values for P/S were 1.04 ± 0.10 and 1.15 ± 0.09 and for A/P were 1.11 ± 0.20 and 1.43 ± 0.11, respectively. Based on the non-significantly different values obtained for the samples before and after POA treatment, it was assumed that the relative fatty acid did not change significantly after solvent mixture treatment. Moreover, the dissolution of the treated samples was tested by weighing the samples before and after the POA treatment. It was found that the samples did not noticeably dissolve during contact with the POA mixture.

Based on these results, it can be concluded that influence of the POA mixture on the important parts of paintings is not significant. However, it must be emphasized that before using the pNIPA-LAP organogel on any work of art, typical preliminary safety tests that are conducted in conservation practice should be performed.

### 3.5 Tests of the pNIPA-LAP Organogel on an Oil Paintings Conserved by the Dutch Method

Finally, a test of the usability of the pNIPA-LAP organogel on a real work of art was carried out. To this end, the painting named “Zinnias in a Blue Vase” (Polish painter, ca. 1930, oil, canvas size 53 × 43 cm, private collection), which was lined using the Dutch method on a rigid substrate around 1950, was selected. Before the treatment, the influence of the POA cleaning mixture on the paint was tested. A small piece of the face of the painting was exposed to POA, and neither discoloration of the cleaning mixture nor changes in the painting was observed. After that, it was decided to start the process of removing the wax resin adhesive.

**Figure 9:**
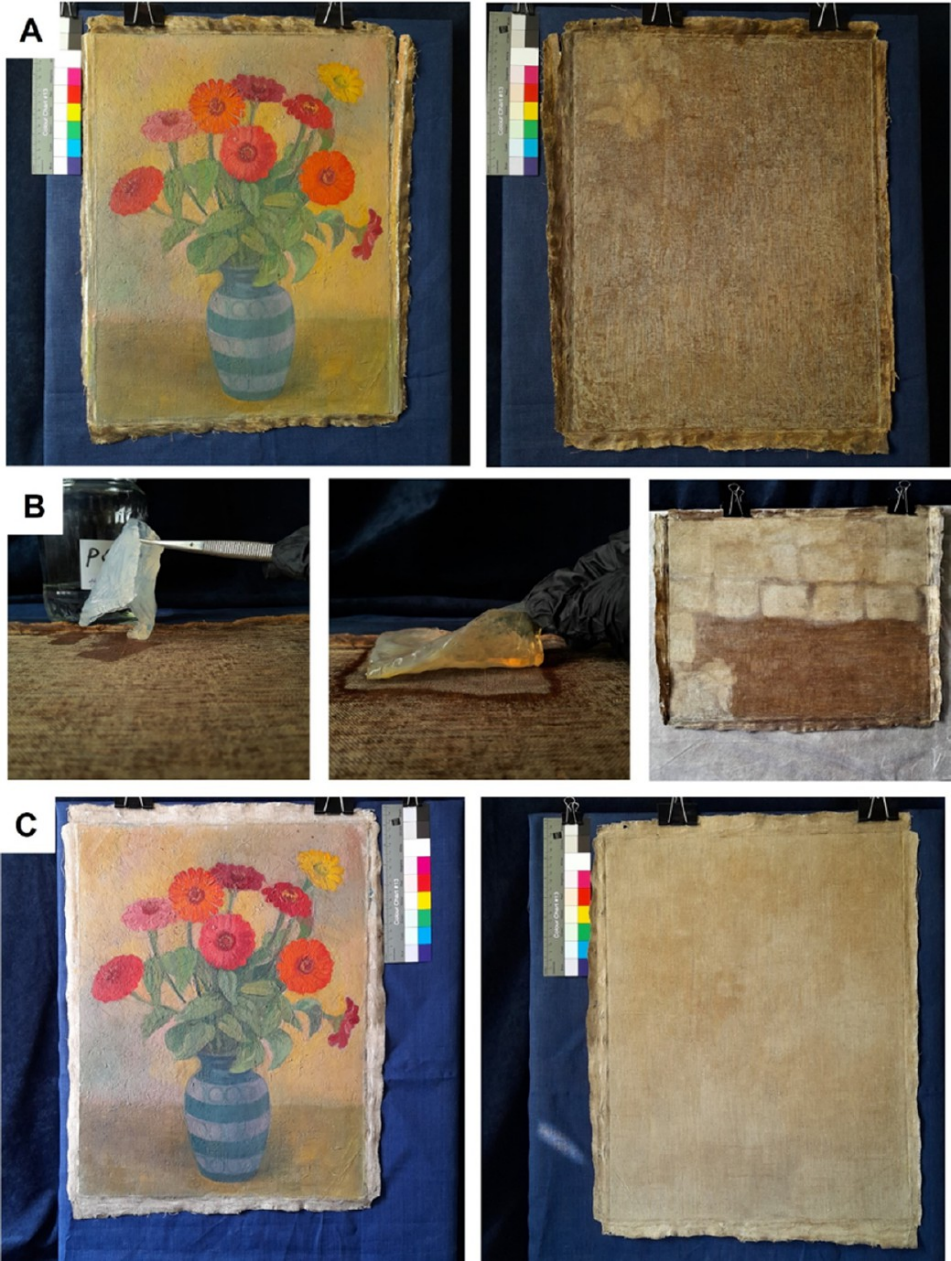
Results of a test removal of wax resin adhesive from the reverse of the oil painting “Zinnias in a Blue Vase” using the pNIPA-LAP-POA organogel: view of the face and the reverse of the painting before cleaning (A), subsequent stages of applying the pieces of organogel on the reverse of the painting (B), and view of the face and the reverse of the painting after cleaning (C).

The extraction of wax resin adhesive from the reverse of the painting was preceded by the mechanical removal of the secondary rigid lining substructure and the excess wax resin adhesive. The extraction was carried out using a few pNIPA-LAP-POA organogel pieces with varying dimensions, e.g., 9 × 6 × 0.5 cm and 11.5 × 5 × 0.2 cm. The organogel was put on the reverse of the painting, covered with Melinex foil, and loaded with sandbags or a Petri dish to reduce solvent evaporation. The sandbags were used to ensure better contact between the organogel and the canvas. The organogel was applied in two regimes: a single application and rinsing or a double application with turning upside down and rinsing—both methods were effective. The interaction times of the organogel with the canvas were 15, 20, and 30 min. In some cases, it was necessary to apply the organogel more than once in the same place to obtain satisfactory results. A positive result was also obtained when repeatedly rinsing in the POA mixture, which allowed the amount of the cleaning mixture consumed to be reduced (for the cleaning, 1 L of POA mixture was used). During the work, the condition of the face of the painting was checked regularly. The wax resin adhesive was completely removed from the reverse of the painting, even from the hollows between the weaves of the canvas, leaving the clean canvas ready for relining. Importantly, as a result of removing the wax resin adhesive, outstanding changes in the appearance of the face of the painting were also observed, which regained its shine and colors. The painting “Zinnias in a Blue Vase” before and after cleaning and the cleaning process are shown in Figure 9A,C. As can be seen, the use of this organogel gave excellent results. The canvas cleaned using the pNIPA-LAP organogel was next successfully subjected to a relining procedure using a modern transparent lining material: BEVA 371 film and glass fiber fabric.^47,48^

**Figure 10:**
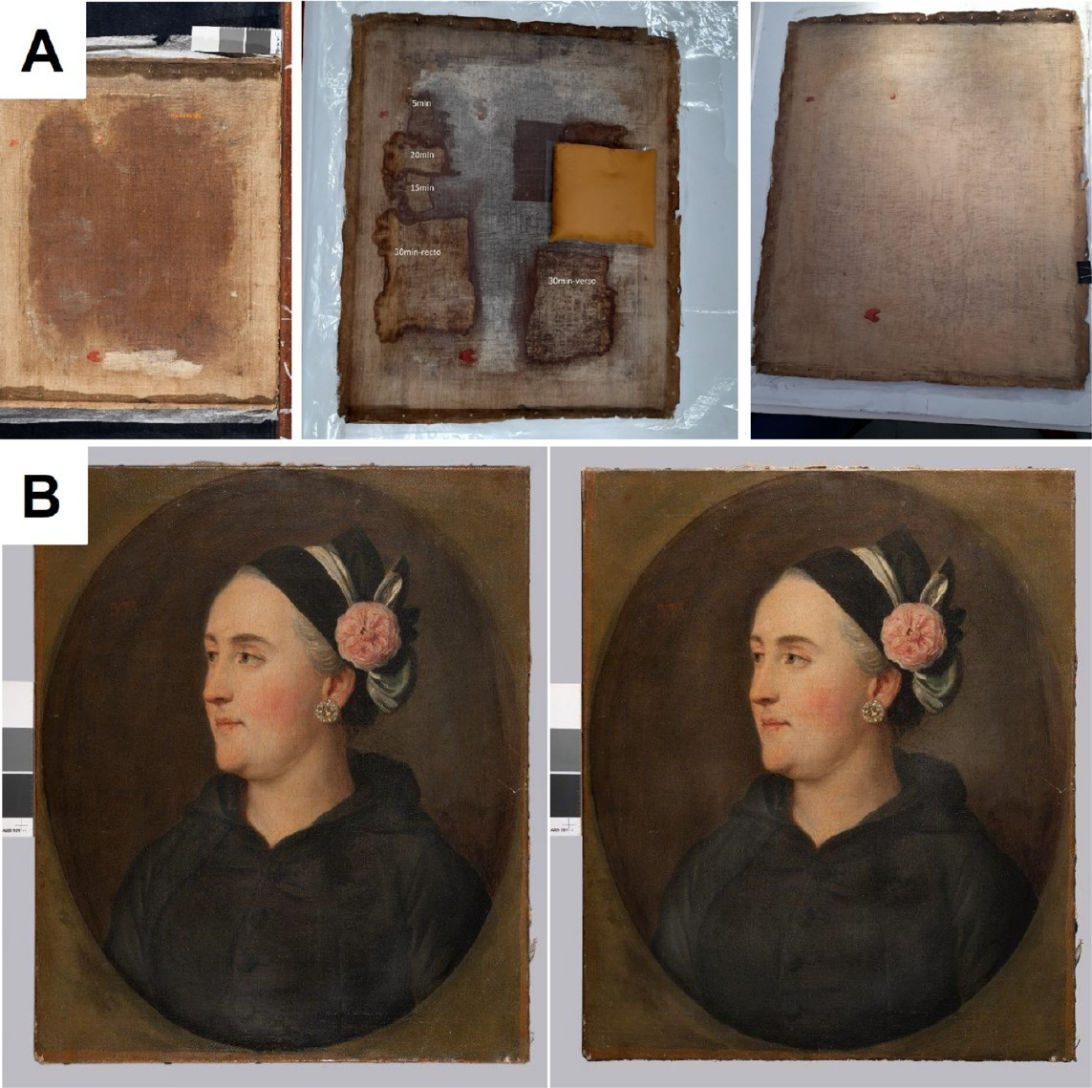
Results of test removal of wax resin adhesive from the reverse of the oil painting “Tsarina Catharina” using the pNIPA-LAP-POA organogel: view of the reverse of the painting before, during, and after cleaning (A) and view of the face of painting before (right) and after cleaning (left) (B).

For the second restoration test, the older painting was selected “*Tsarina Catherine”* dated to the 18th century, the author is unknown as well as the exact date of restoration with the wax-resin. The cleaning process was analogous to that described in the above example. Figure 10 A shows the cleaning steps, from the right side: the canvas before pNIPA-LAP-POA treatment, in the middle canvas during organogel treatment (adjusting the cleaning time), and at the end canvas cleaned from wax resin adhesive. In the opinion of conservators performing the treatment, the face of painting regained its original color after extraction and cleaning with pNIPA-LAP-POA, see Figure 10B; even though it is not as good visible as on example presented in Figure 9 due to dark color of painting. The expertise included the following advantages of the presented solution: (1) after applying the organogel, the wax resin mass was removed from the canvas reverse of the painting to the extent that it would be possible to use modern synthetic resins as an impregnation instead; (2) the method with the use of organogel allowed for the procedure to be carried out in a common room (conservation studio), in a manner that did not conflict with other conservation works; and (3) the interval system of applying the gel allows for other conservation works to be performed simultaneously, which made the treatment less time-consuming. The conservators found this method easy to use, safe for the conservator and, after previous tests, also safe for the painting. To conclude, the extraction procedure performed with the use of the organogel allowed for the removal of the wax resin mass from the back of the painting, from the collection of the National Museum in Warsaw, Nieborów Palace branch, to the extent expected, allowing for the replacement of the destructive mass with a synthetic resin impregnation of good quality. Now, “*Tsarina Catherine*” painting is available to visitors at The Palace on the Isle in Warsaw’s Royal Baths Park.

It should also be mentioned that it is important to control the amount of solvent mixture released into the artwork. It is known that in some cases, deep penetration of solvents is indicated/needed, while in others, only surface action is required. The amount of the solvent mixture released from the organogel depends on several factors, e.g., time of contact, surface textures, canvas thickness, temperature, and adhesive composition. It must be emphasized that before using the organogel on any work of art, preliminary tests should be performed to optimize this parameter. The amount of solvent mixture released can be controlled by the contact time and the amount of solvent mixture in the organogel (swelling ratio).

## 4 Conclusions

We developed novel mixtures of solvents and materials for the extraction of wax resin lining adhesive from the reverse of paintings. Based on the analysis of Teas’ parameters, the ability of mixtures to simultaneously dissolve waxes and organic resins and inertia toward the polymerized oils present in the painting was predicted. Among various compositions, due to economic and safety reasons, one that contains isopropanol (35%, v/v), isooctane (45%), and acetone (20%) was selected for more detailed investigation. The selected cleaning mixtures were immobilized in a polymeric gel carrier exhibiting amphiphilic properties based on poly(*N*-isopropylacrylamide) cross-linked with an inorganic nanostructure Laponite XLS. The nanocomposite organogel obtained exhibits many useful properties, i.e., high cleaning mixture content, high elasticity, cohesiveness, and mechanical strength, thanks to which the gel carrier does not undergo mechanical degradation either during the process of removing wax resin lining adhesive or during the regeneration. We found that a single (more than 20 min) local application of the organogel soaked in the cleaning mixture allows for excellent cleaning of the canvas of wax resin lining adhesive. The short treatment time is highly beneficial because it allows the exposure time of the painting’s support to the cleaning agents to be limited. In addition, the excellent mechanical strength of the organogel enables works of art to be cleaned without leaving any residual pieces of the gel matrix on the cleaned surface. The durability and mechanical flexibility of the organogel allow it to be used during the extraction of wax resin lining adhesive even from the hollows between the weaves of the canvas. The organogel can be used many times with no visible loss of cleaning ability. Moreover, the material is reusable. The proposed method has an effectiveness comparable with the effectiveness of classical extraction using TCE while providing safety for the work of art and reducing harmfulness to humans.

The organogel is safe for canvas and painting when used on the reverse of paintings because it is neutral to cellulose components as well as for the polymerized oils contained in paint layers, which has been confirmed in conservation tests as well as FTIR and GC–MS measurements. After cleaning with the organogel, the paintings regain their shine and color, while the reverse of the paintings is ready, without further preparation, for applying a new conservation approach. The two original oil painting “*Zinnias in a Blue Vase*” from 1930, artist unknown, painting from private collection, and “*Tsarina Catherine*” artist unknown, painting in the collection of the National Museum of Warsaw, were successfully renovated with the usage of nanocomposite organogel filled with the mixture of organic solvents pNIPA-LAP-POA.
